# Short-term S100A8/A9 Blockade Promotes Cardiac Neovascularization after Myocardial Infarction

**DOI:** 10.1007/s12265-024-10542-6

**Published:** 2024-07-15

**Authors:** Razvan Gheorghita Mares, Viorel Iulian Suica, Elena Uyy, Raluca Maria Boteanu, Luminita Ivan, Iuliu Gabriel Cocuz, Adrian Horatiu Sabau, Vikas Yadav, Istvan Adorjan Szabo, Ovidiu Simion Cotoi, Mihaela Elena Tomut, Gabriel Jakobsson, Maya Simionescu, Felicia Antohe, Alexandru Schiopu

**Affiliations:** 1 Department of Pathophysiology, George Emil Palade University of Medicine, Pharmacy, Science, and Technology of Targu Mures, Targu Mures, Romania; 2 Department of Proteomics, Institute of Cellular Biology and Pathology “Nicolae Simionescu”, Bucharest, Romania; 3 Clinical County Hospital, Targu Mures, Romania; 4 Department of Clinical Sciences, Lund University, Malmö, Sweden; 5 Molecular and Cellular Pharmacology – Functional Genomics, Institute of Cellular Biology and Pathology “Nicolae Simionescu”, Bucharest, Romania; 6 Department of Translational Medicine, Lund University, Malmö, Sweden; 7 Department of Internal Medicine, Skane University Hospital, Lund, Sweden

**Keywords:** S100A8/A9, Myocardial infarction, Inflammation, Neovascularization

## Abstract

Acute-phase inhibition of the pro-inflammatory alarmin S100A8/A9 improves cardiac function post-myocardial infarction (MI), but the mechanisms underlying the long-term benefits of this short-term treatment remain to be elucidated. Here, we assessed the effects of S100A8/A9 blockade with the small-molecule inhibitor ABR-238901 on myocardial neovascularization in mice with induced MI. The treatment significantly reduced S100A9 and increased neovascularization in the myocardium, assessed by CD31 staining. Proteomic analysis by mass-spectrometry showed strong myocardial upregulation of the pro-angiogenic proteins filamin A (~ 10-fold) and reticulon 4 (~ 5-fold), and downregulation of the anti-angiogenic proteins Ras homolog gene family member A (RhoA, ~ 4.7-fold), neutrophilic granule protein (Ngp, ~ 4.0-fold), and cathelicidin antimicrobial peptide (Camp, ~ 4.4-fold) versus controls. In-vitro, ABR-238901 protected against apoptosis induced by recombinant human S100A8/A9 in human umbilical vein endothelial cells (HUVECs). In conclusion, S100A8/A9 blockade promotes post-MI myocardial neovascularization by favorably modulating pro-angiogenic proteins in the myocardium and by inhibiting endothelial cell apoptosis.

**Graphical Abstract:**

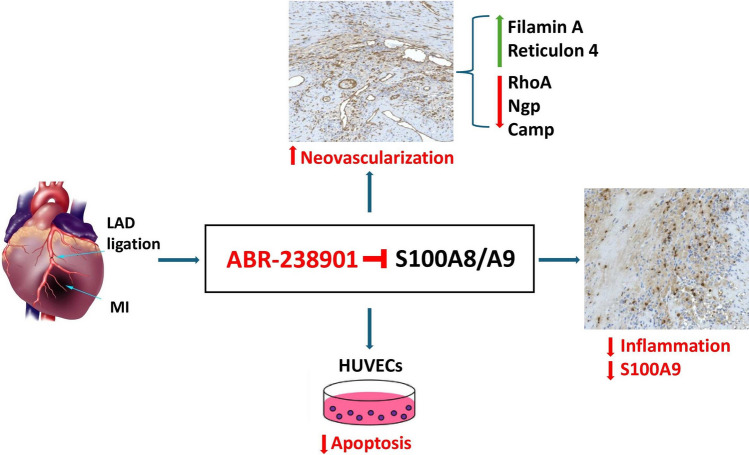

**Supplementary Information:**

The online version contains supplementary material available at 10.1007/s12265-024-10542-6.

## Introduction

Myocardial infarction (MI) triggers a robust inflammatory reaction through immune mechanisms. This early inflammatory response is vital for long-term cardiac repair, as it mediates the clearance of dead cardiac tissue [1]. However, an excessive or sustained inflammatory response enhances cardiac damage and promotes adverse ventricular remodeling and heart failure [2]. The necrotic cardiac tissue releases proteins that signal tissue injury, known as danger-associated molecular patterns (DAMPs) or alarmins [3]. DAMPs such as S100 proteins, high mobility group box-1 (HMGB1) or heat-shock proteins (HSPs) are the most studied in this context [4]. Following myocardial ischemia, S100A8/A9 is rapidly released in the myocardium and circulation, mainly from activated neutrophils and monocytes/macrophages, and promotes recruitment and activation of innate immune cells by engaging its cognate receptors TRL4 and RAGE [4–6]. The genes encoding S100A8 and S100A9 are the most upregulated genes in the heart in the immediate post-MI period and the S100A8/A9 heterodimer is highly increased in MI patient plasma, suggesting important roles of the protein in the acute phase of MI [6, 7]. We have previously shown that S100A8/A9 blockade administered for three days during the acute post-MI phase inhibits systemic and myocardial inflammation, reduces myocardial injury and improves long-term cardiac function [8].

Myocardial repair requires an intense neo-angiogenic response to increase nutrient and oxygen delivery in the ischemic area and border zone [9, 10]. Enhanced angiogenesis post-MI contributes to myocardial viability, lowers scar expansion and reduces long-term left ventricular remodeling and dysfunction. Signals released from the ischemic and necrotic tissue are the primary drivers of neoangiogenesis [11]. However, strong inflammation in the myocardium may inhibit these signals and damage the newly formed endothelium. In the current work, we investigated the effects of short-term S100A8/A9 blockade on myocardial neovascularization post-MI and examined the expression of key proteins involved in this process. *In-vitro,* we assessed whether S100A8/A9 has a direct pro-apoptotic effect on endothelial cells, which can be reversed by the treatment.

## Methods

A detailed description of the Methods section is provided in the Supplementary material online.

### Experimental Animals

Male and female wild-type (C57BL/6) mice, 8–12 weeks of age, were purchased from the Cantacuzino National Research and Development Institute, Bucharest, Romania or bread in-house at the animal care facility of the Institute for Cellular Biology and Pathology (ICBP) “N. Simionescu”, Bucharest, Romania. All *in-vivo* experiments for the histology analysis were conducted at “George Emil Palade” University of Medicine, Pharmacy, Science, and Technology of Targu-Mures, Romania. Experiments involving proteomic analysis have been performed at ICBP “N. Simionescu”. All experimental procedures have been approved by the ethics commitees of the respective institution, according to the relevant Romanian laws.

### Experimental Groups and Treatments

MI was induced by permanent left coronary artery (LCA) ligation, as previously described [12, 13]. Immediately after the MI surgery, the mice were randomly assigned into 2 groups and treated with PBS (MI group) or with 30 mg/kg of the small-molecule S100A8/A9 blocker ABR-238901 (ABR) diluted in PBS (MI + ABR group). ABR-238901, a gift from Active Biotech AB (Lund, Sweden), inhibits the binding of S100A8/A9 to its receptors [8]. Separate mouse groups were sacrificed at 1-, 3- and 7-days post-MI and the hearts were collected for immunohistochemical analysis. Mice harvested on day 1 post-MI received one i.p. injection of either PBS or ABR, administered immediately after MI. All other mouse groups received a total of three i.p. injections of PBS or ABR administered at the time of the MI, and repeated after 24 and 48 h. For the proteomics analysis, we harvested the infarcted regions of the cardiac left ventricle below the ligature at 7-days post-MI and homogenized the tissue with TRIzol Reagent (Sigma-Aldrich, MO, USA) for subsequent protein extraction. The number and sex of the mice included in the final MI mouse groups in all experiments are specified in the figure legends.

### Immunohistochemistry

The expression of S100A9 and of the endothelial cell marker CD31 in cardiac tissue was assessed by immunohistochemical staining at 1, 3 and 7 days post-MI. We used a monoclonal IgG rabbit anti-mouse anti-S100A9 primary antibody (clone D3U8M) or a monoclonal IgG rabbit-anti mouse anti-CD31 primary antibody (PECAM-1, clone D8V9E) (Cell Signaling Technology, Danvers, MA, USA), followed by detection with the BrightVision Goat-Anti Rabbit IgG (H + L)-Poly-HRP Biotin-free secondary antibody (Immunologic, Amsterdam, The Netherlands). All images were analysed by QuPath version 0.3.0 (https://qupath.github.io). The S100A9 and CD31 presence was quantified as a percentage of the entire left ventricle (LV) area, and of the infarcted/border zone and the remote myocardial area separately.

### Liquid Chromatography – Tandem Mass Spectrometry (LC‐MS/MS) and Statistical Analysis

The proteomic analysis was performed by liquid chromatography and mass spectrometry as previously described [14]. From each experimental condition (Sham, MI and MI + ABR), 50 μg of proteins were extracted from the infarcted regions of cardiac left ventricle below the ligature (MI groups) or from the whole heart (Sham group). The mass spectrometry raw data were analyzed with the Proteome Discoverer 2.4 software (Thermo Scientific), and the UniProtKB/Swiss‐Prot mouse reference protein database (UP000000589 Proteome ID, v.04.2019) was used for protein inference. The target protein false discovery rate (FDR) was set below 0.05. ANOVA hypothesis test was used to determine the statistical significance, followed by the Benjamini–Hochberg FDR-based correction. We selected only the proteins that were significantly up- or down- regulated by > 1.25-fold in the MI + ABR/MI comparison. We used the “Biological processes” Gene Ontology analysis to reveal the over-represented angiogenesis-related biological processes, with a Benjamini–Hochberg FDR-based corrected *p*-value < 0.05.

### Endothelial Cell Apoptosis Assay In-vitro

Human Umbilical Vein Endothelial Cells (HUVECs, Thermo Fisher Scientific, Waltham, MA) were cultured in endothelial cell basal medium (Promocell GmbH, Heidelberg, Germany) containing 2% serum, growth supplements (human recombinant epidermal growth factor and fibroblast growth factor) and 1% antibiotics (Invitrogen, CA, USA). The cells were seeded into 96-well plates at 3 × 10^4^ cells per well and allowed to adhere overnight. Thereafter, the cells were treated with 5 μg/ml or 10 μg/ml recombinant human S100A8/A9 in the presence or absence of 100μM ABR-238901 for 24 h in 0.5% low serum medium. Untreated cells and cells treated with 100μM ABR-238901 alone served as controls. To assess apoptosis induction, we measured the levels of active caspase-3/7 in cell lysates by using the Caspase-Glo 3/7 kit (Promega, Wisconsin, USA).

### Statistical Analysis

All data from the immunohistochemistry experiments are expressed as mean ± standard deviation (SD). The Shapiro–Wilk test confirmed the normality of data distribution for groups with low numbers of replicates, allowing parametric tests to be used for the statistical analysis. Comparisons among three groups were performed using one-way ANOVA with Fisher’s LSD post-hoc test. Comparisons between two groups were performed with Student’s T-test. The GraphPad Prism 6.0 software (GraphPad, CA, USA) was used for the statistical analysis. A *p*-value < 0.05 was considered to be statistically significant.

## Results

### S100A8/A9 Blockade Lowers S100A9 Expression in the Infarcted and Remote Myocardium post-MI

**Fig. 1:**
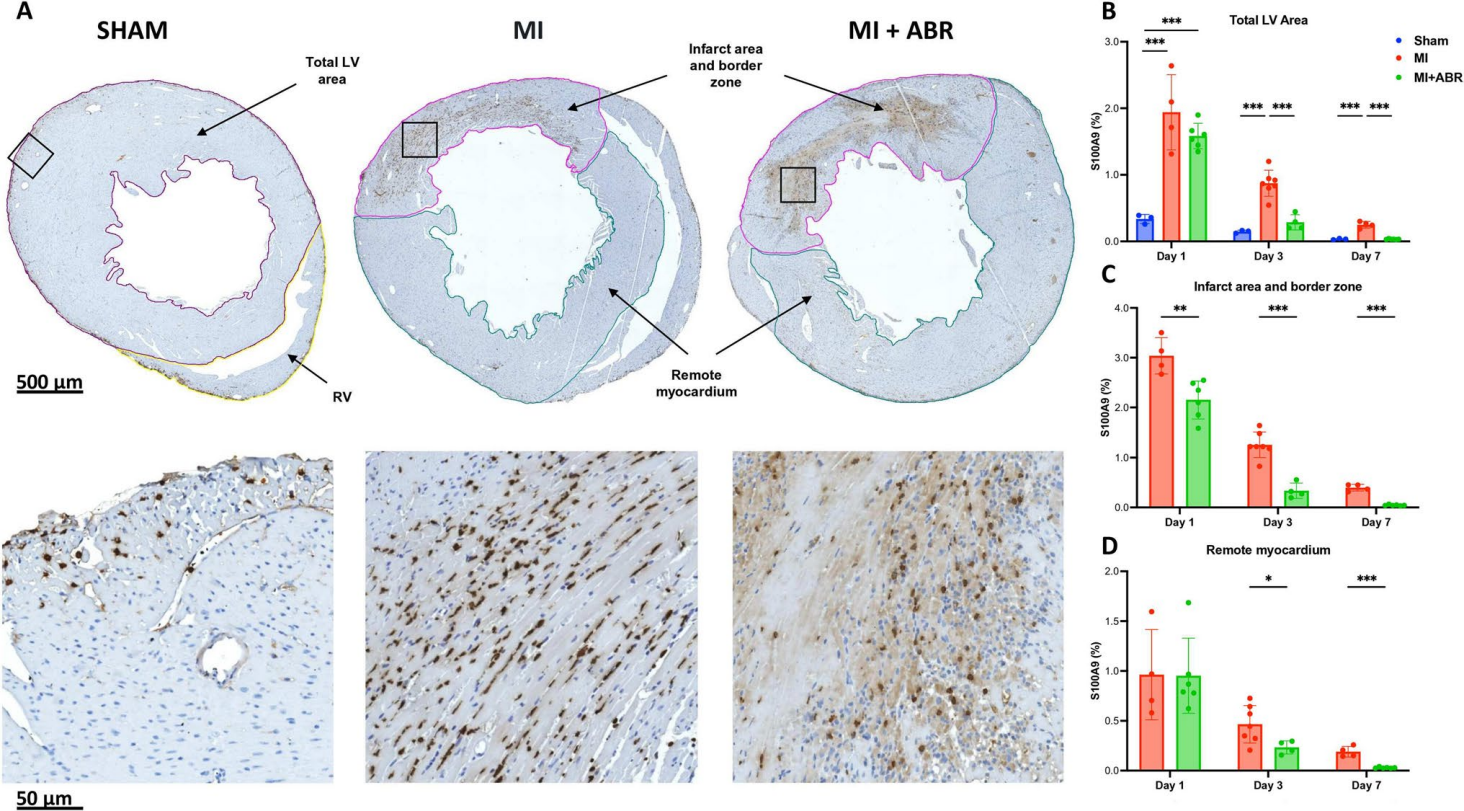
Short-term ABR-238901 treatment significantly reduces S100A9 levels in the myocardium after MI. (**A**) S100A9 staining in the three groups on day 3 post-MI. The images in the lower part of the figure are enlarged views of the myocardial areas marked by the black squares. S100A9 infiltration was measured at 1-day (Sham, *n* = 3; MI, *n* = 4; MI + ABR, *n* = 6; females), 3-days (Sham, *n* = 3; MI, *n* = 7; MI + ABR, *n* = 4; females), and 7-days post-MI (Sham, *n* = 3; MI, *n* = 4; MI + ABR, *n* = 6; all females) in the total LV area (**B**), infarct area and border zone (**C**) and remote myocardium (**D**). Data are presented as mean ± SD. *p < 0.05, **p < 0.01, ***p < 0.005. MI + ABR, mice with MI treated with ABR-238901 for up to 3 days post-MI

We assessed the dynamics and localization of S100A9 infiltration post-MI in mice treated with PBS and ABR-238901. Sham-operated animals served as controls. S100A9 was highly expressed on day 1 post-ischemia in the PBS-treated MI mice, mainly in the infarcted and border areas, and gradually decreased to day 7 (Fig. 1). Importantly, S100A9 was also elevated in the remote myocardium on day 1 (average 0.96% vs 0.33% in sham controls) showing that the myocardial ischemia induces an inflammatory response of lower intensity in the non-ischemic myocardium as well (Fig. 1B, D).

One ABR-238901 dose significantly lowered S100A9 levels in the infarcted and border zone already on day 1 post-MI (Fig. 1C). The effect was more pronounced on day 3, at the end of the treatment, and the difference between the treatment groups remained significant up to at least day 7 (Fig. 1C). Consequently, ABR-treated mice had very low levels of the protein in the LV on day 7 compared to controls (Fig. 1B). In the remote myocardium, S100A9 was significantly lower in ABR-treated mice compared to PBS controls on days 3 and 7, demonstrating protective anti-inflammatory effects of the treatment in this area as well (Fig. 1D).

### S100A8/A9 Blockade Promotes post-MI Neovascularization

In order to assess whether the previously-demonstrated beneficial long-term effects of the treatment can be explained by improvements of post-ischemic myocardial perfusion, we compared myocardial vascularization in sham-operated hearts and in hearts with MI, with and without ABR-238901 treatment, at 1-, 3- and 7-days post-MI. Capillary bed density was expressed as percentage endothelial CD31-positive area out of total LV area and of the ischemic and remote myocardium separately.

**Fig. 2:**
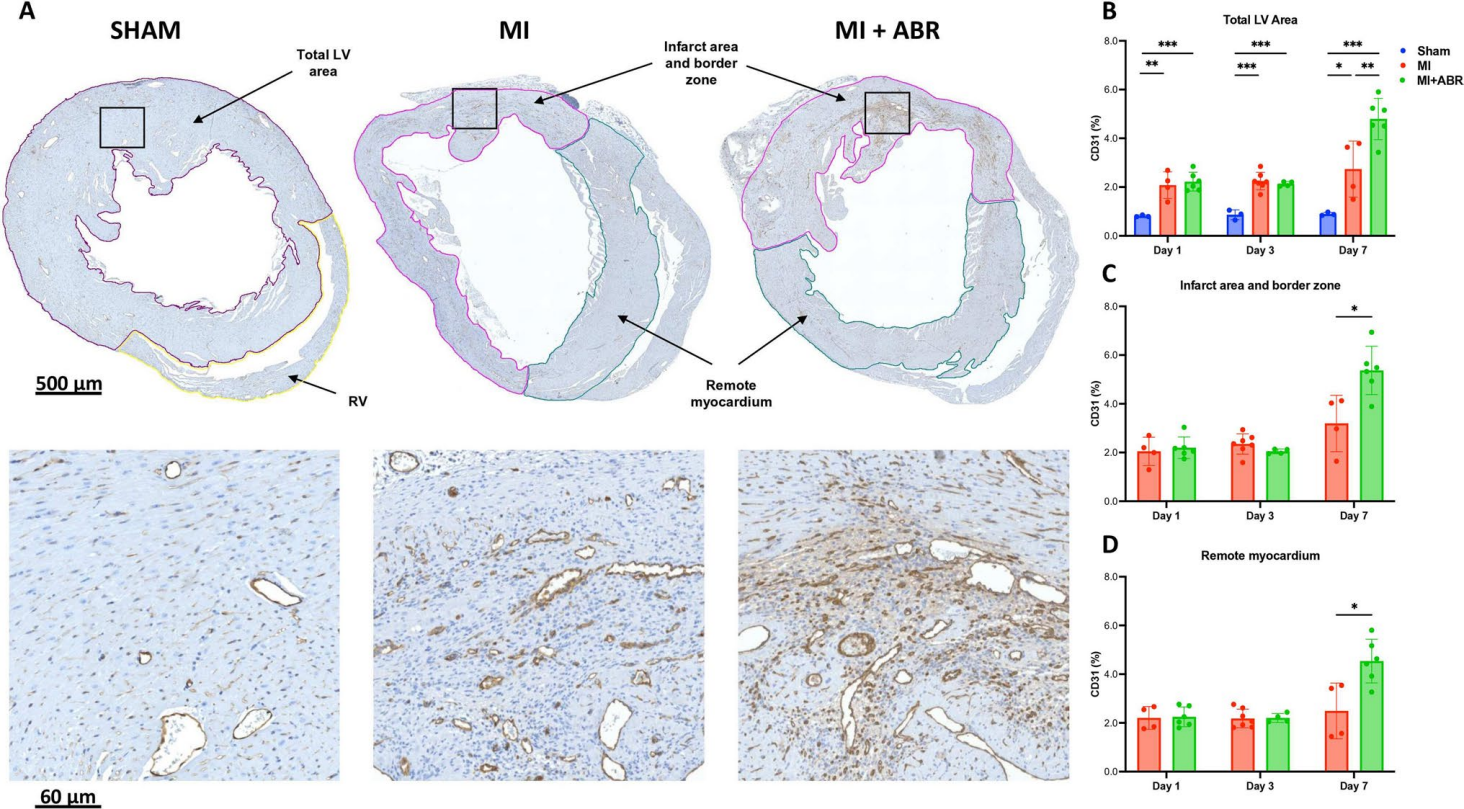
S100A8/A9 blockade induces enhanced neovascularization on day 7 post-MI. (**A**) CD31 staining in the three groups on day 7 post-MI. The images in the lower part of the figure are enlarged views of the myocardial areas marked by the black squares. The bar graphs show the CD31-positive area expressed as percent of the myocardial area at 1-day (Sham, *n* = 3; MI, *n* = 4; MI + ABR, *n* = 6; all females), 3-days (Sham, *n* = 3; MI, *n* = 7; MI + ABR, *n* = 4; all females) and 7-days post-MI (Sham, *n* = 3; MI, *n* = 4; MI + ABR, *n* = 6; all females) in the total LV area (**B**), infarct area and border zone (**C**) and remote myocardium (**D**). Data are presented as mean ± SD. *p < 0.05, **p < 0.01, ***p < 0.005. MI + ABR, mice with MI treated with ABR-238901 for up to 3 days post-MI

In the PBS-treated MI mice, we observed an increase in CD31 staining from day 1 to day 7 throughout the myocardium, compared to sham-operated mice (Fig. 2B). S100A8/A9 inhibition did not influence myocardial vascularization on days 1 and 3 post-MI (Fig. 2B-D). However, the treatment led to a 2.5-fold increase of the vascular area from day 1 to day 7, compared to only 1.6-fold increase in PBS-treated MI mice. On day 7, the capillary bed was significantly more abundant in MI mice treated with ABR-238901 compared with PBS-treated MI mice, in both the infarcted area and in the remote myocardium (Fig. 2A-D).

**Fig. 3:**
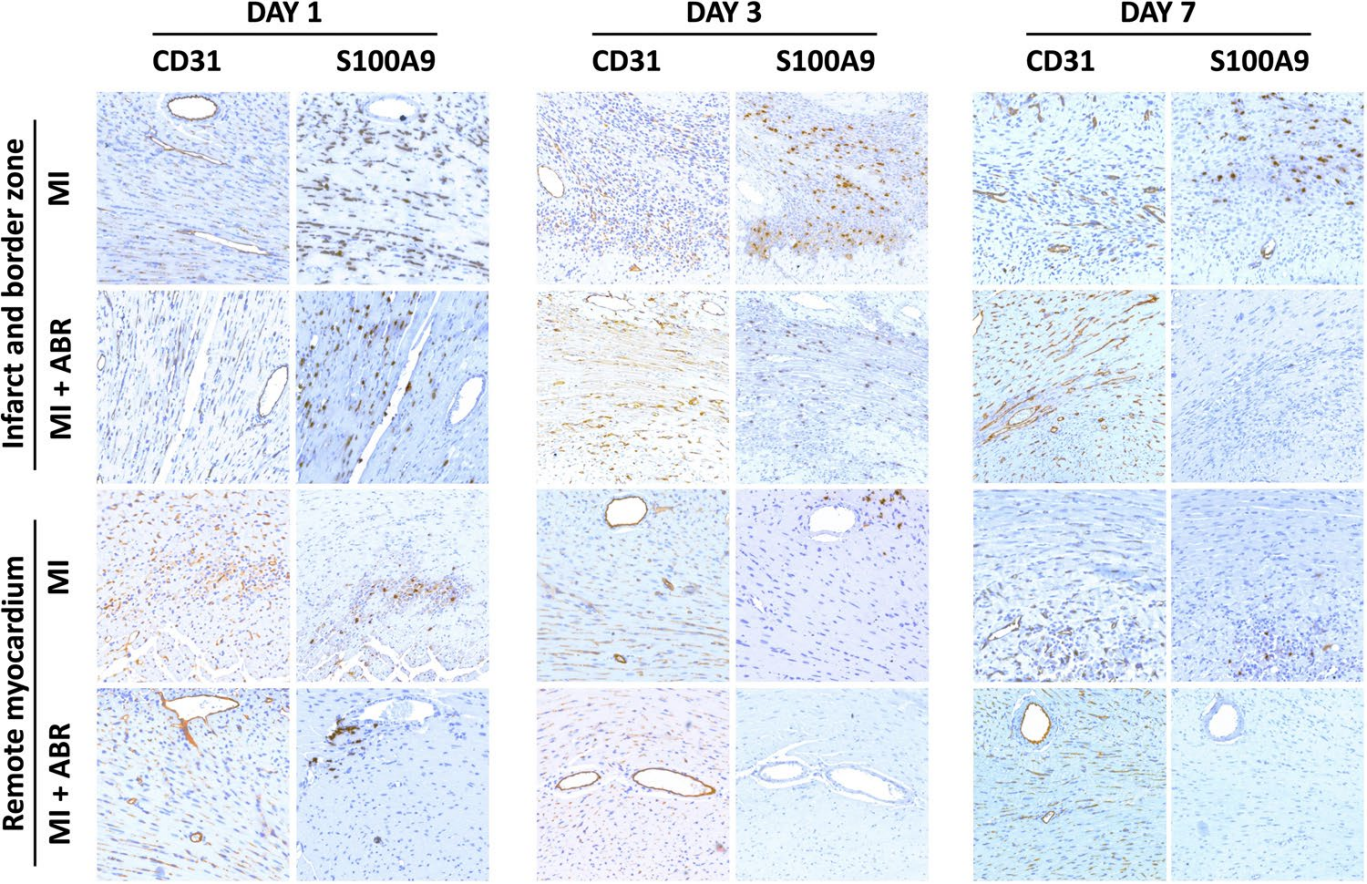
Side-by-side comparison of S100A9 infiltration and CD31-positive vasculature in the myocardium. Comparison of S100A9-positive and CD31-positive staining in the same myocardial areas from the infarcted and remote myocardium, in mice with induced MI treated with PBS or ABR-238901 and harvested on days 1, 3 and 7 post-MI. MI + ABR, mice with MI treated with ABR-238901 for up to 3 days post-MI

A side-by-side comparison of CD31 and S100A9 staining in the same LV areas shows lower S100A9 infiltration and a rapid decrease of S100A9 tissue presence in ABR-treated mice, in parallel with an increase in CD31-positive vasculature (Fig. 3). The effects were most prominent in the infarcted myocardium, but could also be observed in remote areas. This qualitative assessment might indirectly support a potential inhibitory role of S100A9 or of S100A9-positive cells against myocardial neoangiogenesis post-MI, which is reversed by ABR-238901.

### Angiogenic Mediators and Processes Modulated by S100A8/A9 Inhibition

**Fig. 4:**
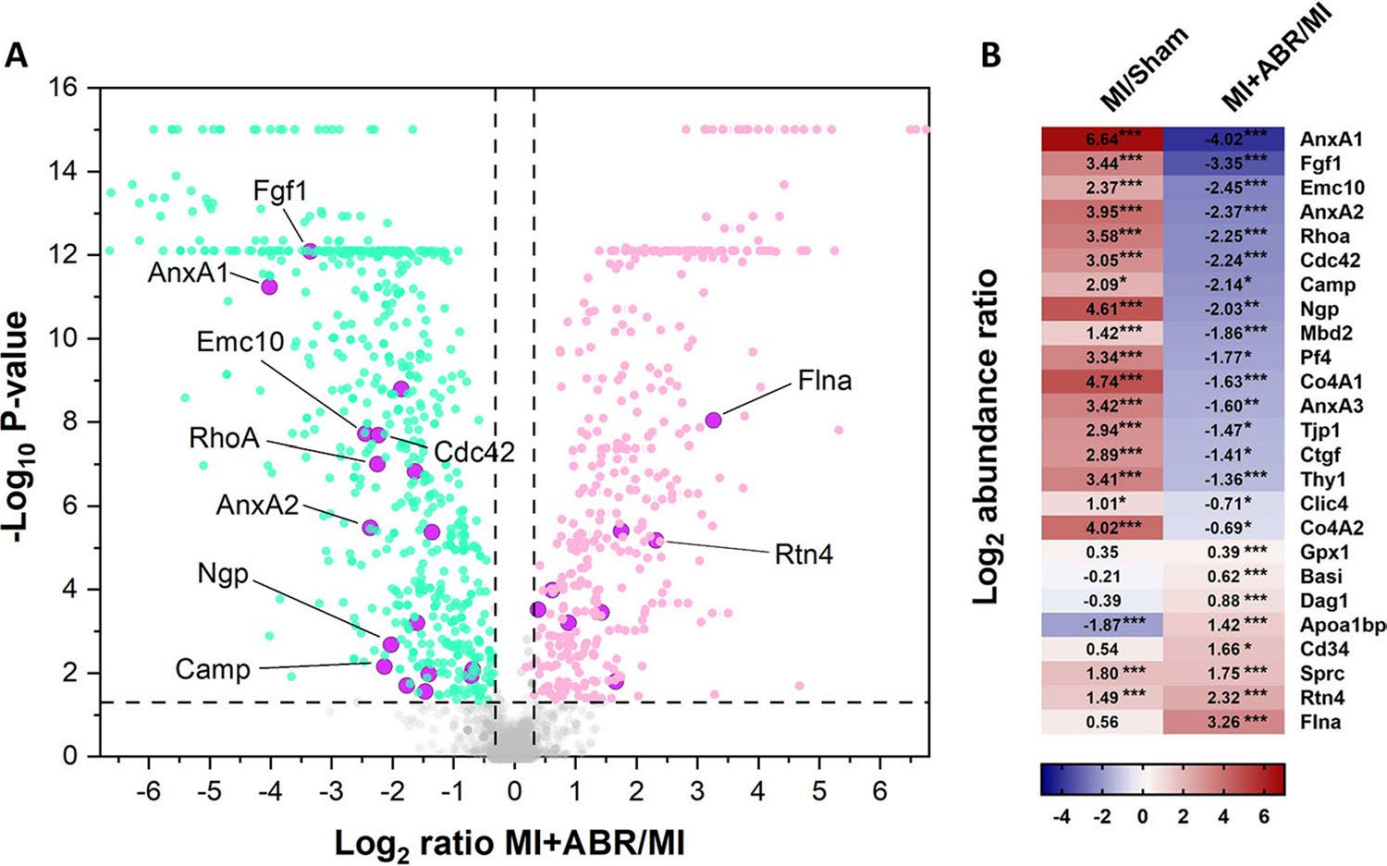
S100A8/A9 blockade significantly impacts the abundance of angiogenesis-related proteins in the post-ischemic myocardium. Differential proteomic analysis of infarcted LV from the PBS-treated (*n* = 4; 2 females and 2 males) and the ABR-treated (*n* = 5; 3 females and 2 males) MI groups. (**A**) Volcano plot depicting the distribution of differentially abundant proteins, separated by fold change and adjusted *p*-value. The green dots are significantly down-regulated proteins (ratio of medians MI + ABR/MI < 0.8, *p* < 0.05) and the pink dots are significantly up-regulated (ratio of medians MI + ABR/MI > 1.25, *p* < 0.05). Proteins associated with angiogenesis are marked by purple circles. Proteins upregulated or down-regulated by more than fourfold are annotated with the gene name. The *p*-values were obtained using ANOVA followed by the Benjamini‐Hochberg correction for false discovery rate. (**B**) Heatmap representation of differentially expressed proteins involved in angiogenesis-linked biological processes between the PBS-treated MI (*n* = 4) and sham (*n* = 3) groups, and between the ABR- and PBS-treated groups, respectively. The colour scale indicates the regulation level between the groups (red represents up-regulation and blue down-regulation). DEPs were considered to be significant at an adjusted *p*-value < 0.05. *p < 0.05, **p < 0.01, ***p < 0.001

In order to define the underlying pathways and mediators leading to the observed effects of S100A8/A9 blockade on coronary circulation, we performed a proteomic analysis of LV extracts by qualitative and relative quantitative mass spectrometry on day 7 post-MI. Comparison of the proteome from the ABR-treated and PBS-treated infarcted myocardium identified 25 proteins implicated in biological processes linked with angiogenesis that were up- or down-regulated > 1.25 times (Fig. 4). The Gene Ontology (GO) analysis revealed the following over-represented processes involving these proteins: angiogenesis (FDR *p* = 1.58 × 10^–09^), regulation of angiogenesis (FDR *p* = 3.22 × 10^–05^), positive regulation of angiogenesis (FDR *p* = 8.54 × 10^–04^), negative regulation of angiogenesis (FDR *p* = 1.16 × 10^–02^), sprouting angiogenesis (FDR *p* = 1.37 × 10^–03^), positive regulation of sprouting angiogenesis (FDR *p* = 1.12 × 10^–02^), regulation of sprouting angiogenesis (FDR *p* = 4.32 × 10^–02^) and angiogenesis involved in wound healing (FDR *p* = 1.24 × 10^–02^). The complete list of the identified proteins is presented in the Supplementary material online, Table 1.

**Fig. 5:**
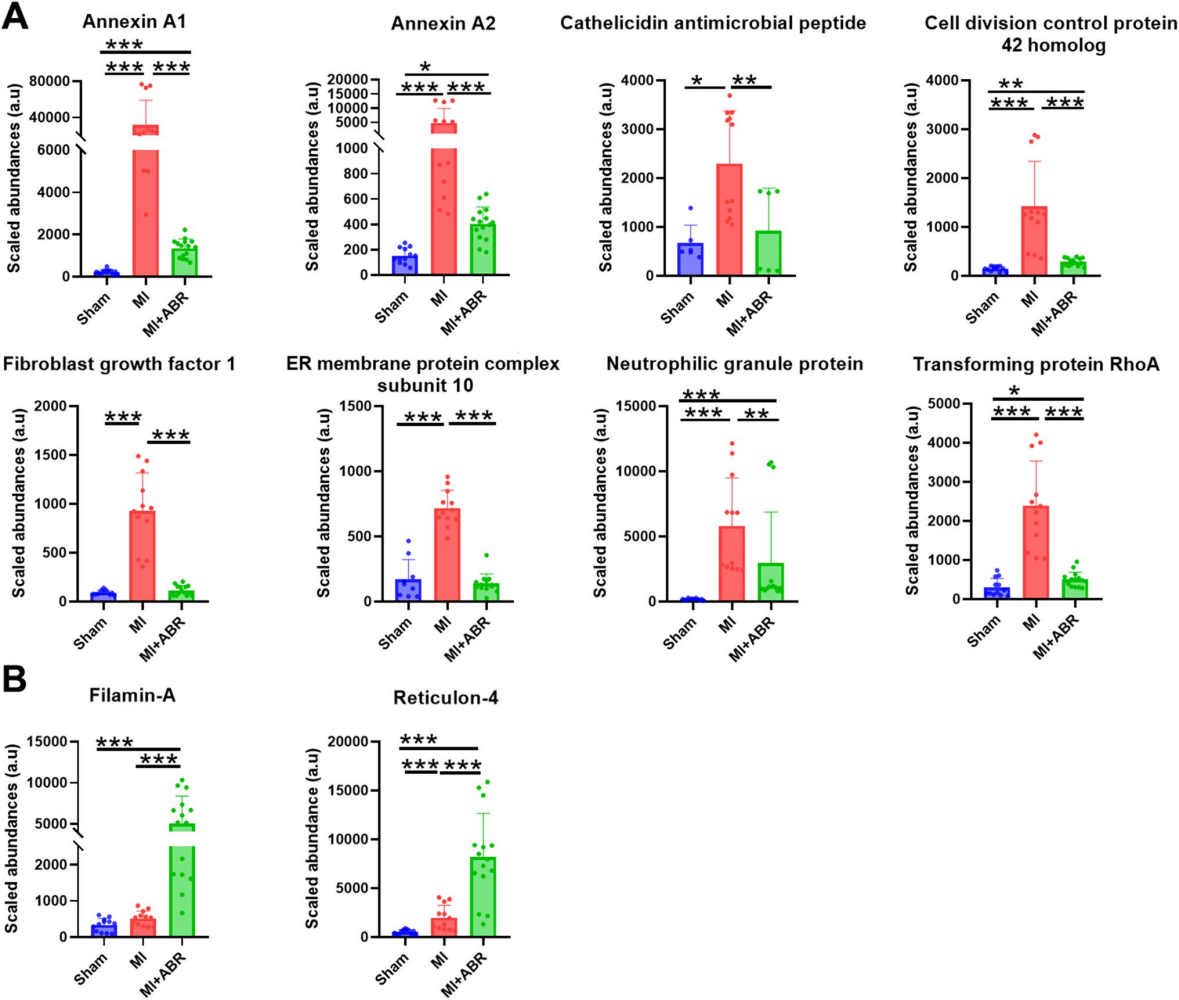
Pro- and anti-angiogenetic proteins modulated by S100A8/A9 blockade in the infarcted myocardium. (**A**) Downregulated proteins and (**B**) upregulated proteins in infarcted LV on day 7 post-MI. Sham, sham-operated control group (*n* = 3 females); MI, mice with MI treated with PBS (*n* = 4; 2 females and 2 males); MI + ABR, MI mice treated with ABR-238901 (*n* = 5; 3 females and 2 males). Cardiac protein extracts from each animal have been analysed in triplicates. The three technical replicates from all the mice are presented on the graphs as individual dots. The columns and error bars represent mean ± SD for each treatment group. *p < 0.05, **p < 0.01, ***p < 0.001

Of the 25 proteins significantly modulated by the treatment, we focused on the 10 proteins that had at least fourfold differences between the MI mice receiving S100A8/A9-blockade and the PBS controls. All these proteins, annotated on Fig. 4A and presented separately in Fig. 5, were upregulated by coronary ischemia. Compared to sham controls, the post-ischemic hearts had an increased abundance of annexin A1 (AnxA1, ~ 100-fold), annexin A2 (AnxA2, ~ 15-fold), fibroblast growth factor 1 (Fgf1, ~ 11-fold), endoplasmic reticulum membrane protein complex subunit 10 (Emc10, ~ fivefold), Ras homolog gene family member A (RhoA, ~ 12-fold), Rho GTPase Cdc42 (Cdc42, ~ eightfold), cathelicidin antimicrobial peptide (Camp, ~ fourfold), neutrophilic granule protein (Ngp, ~ 24-fold), filamin A (Flna, ~ 1.5-fold), and reticulon 4 (Rtn4, ~ 2.8-fold). Of note, the endothelial cell marker CD34 was also increased in ABR-238901-treated mice, further supporting the results of the CD31 quantification (Fig. 4B).

The ABR treatment counteracted the increase of 8 of the 10 proteins compared to the PBS-treated MI group, inducing a significantly lower abundance of Anx1 (~ 16-fold), AnxA2 (~ fivefold), Fgf1 (~ tenfold), Emc10 (~ 5.5 fold), RhoA (~ 4.7-fold), Cdc42 (~ 4.7-fold), Camp (~ 4.4-fold), and Ngp (~ fourfold). In contrast, Flna and Rtn4 were increased by S100A8/A9 blockade by ~ tenfold and ~ fivefold, respectively (Fig. 4B and Fig. 5).

### ABR-238901 Protects Against S100A8/A9-Induced Endothelial Cell Apoptosis

**Fig. 6:**
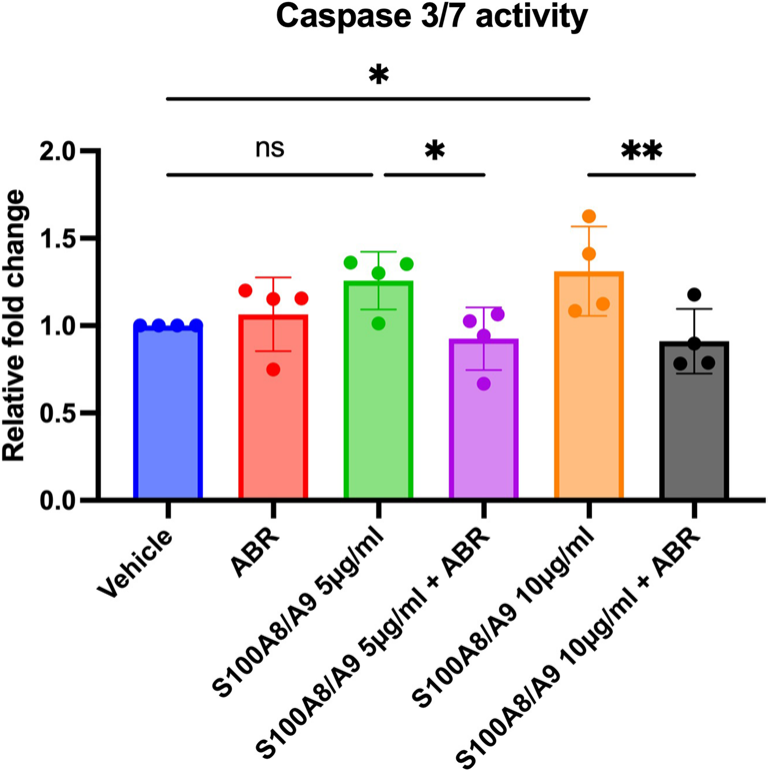
ABR-238901 protects against endothelial cell apoptosis induced by S100A8/A9 in-vitro. Induction of apoptosis was assessed by measurement of caspase-3/7 activity in cultured HUVECs stimulated for 24 h with 5 μg/ml or 10 μg/ml recombinant human S100A8/A9, with or without 100 μM ABR-238901. Caspase-3/7 enzymatic activity was measured in cell homogenates by a chemiluminescent assay. The results are expressed as fold change compared to vehicle-treated control cells. Data are presented as mean ± SD. *p < 0.05, **p < 0.01, ns not significant. ABR, ABR-238901

We have previously shown that S100A8/A9 blockade with ABR-238901 in mice with induced MI favorably modulates the myocardial abundance of a large number of proteins involved in cellular apoptosis [14]. In order to assess whether S100A8/A9 blockade with ABR-238901 may have direct protective effects against endothelial cell apoptosis, we treated HUVECs *in-vitro* with recombinant human S100A8/A9 in the presence or absence of the inhibitor. After 24 h, the degree of cellular apoptosis was assessed by measuring cellular activation of caspase 3/7. We found that S100A8/A9 induced HUVEC apoptosis in a dose-dependent manner, with a 25.7% and 31.2% increase in average caspase activity compared to untreated controls in cells treated with 5 μg/ml and 10 μg/ml S100A8/A9, respectively (Fig. 6). Addition of 100 μM ABR-238901 into the medium prevented endothelial cell apoptosis, regardless of protein concentration (Fig. 6). These results reveal that S100A8/A9, at concentrations similar to the plasma levels present in patients with acute MI [7], has direct apoptotic effects on endothelial cells and may prevent endothelial cell growth and neovascularization in myocardial areas with high S100A8/A9 content. ABR-238901 counteracts these negative effects, which might explain the increased degree of neovascularization in the myocardium of mice with MI treated with the blocker.

## Discussion

Excessive myocardial injury, inflammation, remodeling and fibrosis lead to post-ischemic heart failure and are associated with a negative prognosis in MI patients [15]. At the moment, none of the pharmacologic treatments administered to MI patients specifically address the pathological pathways involved in these processes. We have previously shown that blockade of the pro-inflammatory alarmin S100A8/A9 for three days during the acute post-MI period limits myocardial inflammation and improves cardiac function long-term [8]. However, the mechanisms and pathways responsible for the long-term beneficial effects of this short-term treatment remain to be explained. Here, we show that S100A8/A9 blockade reduces S100A9 presence and increases vascular bed density in both the infarcted and remote myocardium. We also found highly increased abundance of the pro-angiogenic proteins Flna and Rtn4 and lower abundance of the anti-angiogenic proteins RhoA, Ngp, and Camp in the LV of ABR-238901-treated mice compared to PBS-treated controls. Additionally, we demonstrate that S100A8/A9 has a direct dose-dependent apototic effect on endothelial cells in-vitro, which is counteracted by the blocker, and that CD31-positive vessel abundance is lower in myocardial regions with high S100A9 infiltration. These findings are well supported by our previously published data, showing that *in-vivo* S100A8/A9 blockade downregulates pro-apoptotic pathways in the infarcted myocardium [14].

Plasma S100A8/A9 levels correlate with blood neutrophil counts in both healthy individuals [16] and MI patients [17], and the ABR-238901 treatment reduces both circulating neutrophil numbers and plasma S100A8/A9 in mice with MI [8]. These data support the potential use of plasma S100A8/A9 as neutrophil activation marker and as a surrogate biomarker to monitor treatment efficiency. Here, we found that the presence of S100A9 in the myocardium peaked on day 1 post-MI then gradually decreased. This pattern matches the dynamics of neutrophil infiltration, supporting the role of S100A8/A9 as an important neutrophil mediator and biomarker in MI [18, 19]. We and others have previously demonstrated that S100A8/A9 plays an important autocrine role in neutrophil function. S100A8/A9 promotes the pro-inflammatory N1 neutrophil phenotype [20] and stimulates NLRP3 activation and IL-1b production, leading to increased myelopoiesis [21, 22]. Here, we found that S100A9 was expressed most intensively in the infarcted and border area of the left ventricle, but we also noted increased S100A9 presence in the remote myocardium. These results are in accordance with other studies demonstrating elevated inflammation in non-infarcted regions of the heart [23]. We also show that ABR-238901 reduces S100A9 levels in the myocardium, likely due to the reduced neutrophil infiltration [8]. Interestingly, the presence of S100A9 was reduced in both the infarcted and border zone, as well as in the remote myocardium. This effect of the treatment might have important consequences, as lower inflammation in the non-infarcted myocardium might contribute to a lower degree of fibrosis and have a protective effect against the development of diastolic dysfunction [1, 24]. In support of this hypothesis, Yang et al*.* have recently demonstrated significant recruitment of monocytes/macrophages in the remote myocardium of infarcted hearts vs non-infarcted controls, which contributed to LV remodeling. Further, the authors showed that increased T2 values on magnetic resonance imaging (MRI) in the remote myocardium, indicating the presence of inflammation, are associated with long-term LV remodeling [25].

In order to assess the effects of the treatment on myocardial neovascularization, we compared the density of the coronary vascular bed in hearts from healthy controls and mice with MI treated with PBS or ABR-238901. The coronary bed area gradually increased from day 1 to day 7 in the PBS-treated MI mice, which is in line with the previously-described dynamics of post-ischemic myocardial neovascularization [1]. This increase was largely due to enhanced neovascularization of the infarct area and border zone, while vascularization in the remote myocardium remained largely unchanged. Neoangiogenesis was significantly accelerated in ABR-238901-treated mice, where the endothelial cell-positive area reached 4.5% of LV by 7 days post-MI compared to 2.5% in the MI control group. Interestingly, the treatment also increased the degree of vascularization in the remote myocardium. In our previous study, we only found a slight decrease in infarction size in ABR-treated mice, which cannot fully explain the much larger gain in long-term cardiac function [8]. The increased degree of neovascularization demonstrated by the current study may lead to improved cardiomyocyte survival and have an additive beneficial effect on overall cardiac function.

Our proteomics analysis of myocardial tissue on day 7 post-MI identified 25 distinct proteins involved in angiogenesis that were modified by the S100A8/A9 blockade. The treatment increased the abundance of 6 proteins and downregulated 19. The proteins that were upregulated the most, at least 4 times compared to the MI controls, were Flna and Rtn4. The most downregulated proteins were AnxA1, AnxA2, Fgf1, Emc10, the Cdc42, Camp, RhoA, and Ngp.

Flna is an actin-binding protein involved in cell motility. Flna deficiency in endothelial cells leads to impaired endothelial function and neovascularization, increased scar formation and severe cardiac dysfunction post-MI, supporting an important role of the protein in post-ischemic neovascularization and cardiac recovery [26]. In-vitro, Flna-deficient endothelial cells exhibited reduced motility and tubular formation [26]. The reticulon-4 protein family, also known as neurite outgrowth inhibitors (Nogo), are primarily located in the endoplasmic reticulum and consist of three splice variants: Nogo-A, Nogo-B and Nogo-C. In contrast to Nogo-A and Nogo-C, which are mainly found in the central nervous system, Nogo-B and its receptor NgBR are expressed in several organs and play important roles in various biological processes, including angiogenesis [27]. Consistent with our results, Nogo-B was earlier found to be upregulated in the heart following myocardial ischemia, and Nogo-B overexpression in endothelial cells improved neovascularization, reduced scar size and improved cardiac function post-MI through Notch1 signaling [28]. Conversely, Nogo-B inhibition led to endothelial reticulum stress, cardiomyocyte hypertrophy and fibroblast activation in a mouse model of transverse aortic constriction (TAC) [29]. Taken together, these data demonstrate pro-angiogenic, anti-fibrotic and cardioprotective roles for Flna and Nogo-B post-MI and provide mechanistic explanations for the improved vascularization and cardiac function induced by S100A8/A9 blockade.

Among the proteins that were down-regulated by the treatment, the small GTPase RhoA and Ngp have previously been found to have anti-angiogenic effects. Overexpression of constitutively active and wild-type RhoA in endothelial cells prevented in-vivo angiogenesis in mice and inhibited endothelial cell proliferation, migration, and angiogenic sprouting in-vitro [30]. Ngp is a cathelicidin-related protein constitutively expressed in neutrophils that has been shown to inhibit tumor angiogenesis [31]. The role of this protein in the context of MI has not yet been studied. Reduction of Camp levels in the myocardium is another potential protective mechanism induced by our treatment, as Camp has recently been found to promote enhanced myocardial inflammation and injury through TLR4-mediated NLRP3 activation and IL-1β production in neutrophils [32].

Interestingly, S100A8/A9 blockade with ABR-238901 also reduced the abundance of AnxA1, AnxA2, Fgf1, Emc10 and Cdc42, proteins with previously demonstrated pro-angiogenic effects. Annexins are a family of calcium-binding proteins with pleiotropic functions connected to plasma membrane through phospholipid interactions, which can also be found in the cytoplasm or nucleus [33]. Annexins are highly expressed in immune cells but can also be found in other cell types. AnxA1 constitutes 2–4% of neutrophil cytoplasmic proteins and is a potent endogenous anti-inflammatory protein through multiple mechanisms [33]. In addition to its anti-inflammatory properties, AnxA1 was found to promote neo-angiogenesis and cardiac repair post-MI by stimulating VEGF-A secretion from reparatory macrophages [34]. AnxA2 is another member of the annexin family that binds plasminogen and tissue plasminogen activator on the endothelial surface, leading to plasmin activation, fibrinolysis and extracellular matrix proteolysis. Similar to AnxA1, AnxA2 has been found to stimulate angiogenesis [35]. Fgf1 and Emc10 are growth factors primarily produced by monocytes/macrophages that have also been found to promote post-ischemic cardiomyocyte proliferation, myocardial neovascularization and cardiac recovery [36–38]. Finally, pro-angiogenic properties have also been described for the small Rho GTPase Cdc42, a mediator of endothelial cell adhesion, proliferation and differentiation [39]. Although intriguing in the context of the current work, the reduced abundance of these myeloid cell-derived proteins in the myocardium of ABR-238901-treated mice might be explained by the significantly lower numbers of infiltrating neutrophils, monocytes and macrophages, their main producers. As we have shown before, the reduction of circulating and myocardial myeloid cell populations is the main effect of S100A8/A9 blockade in the context of MI [8]. Importantly, we have also shown that extended S100A8/A9 blockade for 21 days post-MI has opposite effects compared to the 3-day treatment, leading to functional impairment and cardiac remodeling [40]. We hypothesize that a sustained reduction of these pro-angiogenic proteins in the myocardium by the long-term treatment might have contributed to impaired neovascularization, remodeling and progressive loss of function in this previous study. This hypothesis requires, however, experimental validation.

### Study Limitations

The Data Dependent Analysis (DDA) technique for mass spectrometry used in the study predominantly identifies high-abundance co-eluting precursors. Consequently, we cannot exclude the possibility that relevant low abundant proteins involved in angiogenesis might have been missed by our assay. Identification of the individual contribution of each protein to the pro-angiogenic and cardioprotective effects of the treatment cannot be discerned by our approach and is not the purpose of the current work. Understanding the relative contribution of each protein would require extensive experiments in mice where these proteins are knocked-out, silenced or overexpressed. As discussed above, support in this direction has already been provided by other groups showing that deficiency of Flna and Nogo-B (Rtn4), the highest upregulated proteins by our treatment, has led to severely impaired cardiac recovery post-MI [28, 31].

## Conclusions

Our results identify short-term S100A8/A9 blockade as an important promoter of cardiac recovery post-MI, by counteracting the pro-apoptotic effects of the alarmin on endothelial cells and by favorably modulating the abundance of several proteins involved in neoangiogenesis. We have previously shown that short-term S100A8/A9 blockade has potent immunomodulatory effects in MI by amplifying anti-apoptotic pathways, inhibiting myocardial damage, and promoting efficient repair and recovery [8, 14]. Our current data reveal that ABR-238901 reduces cardiac infiltration of S100A9-expressing myeloid cells, protects against endothelial cell apoptosis induced by S100A8/A9, and promotes neovascularization. The underlying mechanisms for the pro-angiogenetic effects involve upregulation of Flna and Rtn4, and downregulation of RhoA, Ngp, and Camp. This study further supports our chosen strategy to limit the therapeutic window for S100A8/A9 blockade to the acute inflammatory phase post-MI, and provide important supporting information for the beneficial cardioprotective effects of the treatment.

## Supplementary Information

Below is the link to the electronic supplementary material.

Supplementary file1 (DOCX 44 KB)

Supplementary file2 (DOCX 46 KB)

## Data Availability

The proteomics data have been deposited into the PRIDE [41] partner repository via ProteomeXchange, dataset identifier PXD033683.
