# Supplementary material for: Short-term S100A8/A9 Blockade Promotes Cardiac Neovascularization after Myocardial Infarction
Source: J Cardiovasc Transl Res. 2024 Jul 15;17(6):1389–99. doi: 10.1007/s12265-024-10542-6 (PMC11634919; doi:10.1007/s12265-024-10542-6)
Supplement: Supplementary file 1 — Supplementary file1 (DOCX 44 KB) [file 12265_2024_10542_MOESM1_ESM.docx]

**Short-term S100A8/A9 blockade promotes cardiac neovascularization**

**after myocardial infarction**

Razvan Gheorghita Mares^1^†*, Viorel Iulian Suica^2^†, Elena Uyy^2^, Raluca Maria Boteanu^2^, Luminita Ivan^2^, Iuliu Gabriel Cocuz^1,3^, Adrian Horatiu Sabau^1,3^, Vikas Yadav^4^, Istvan Adorjan Szabo^1^, Ovidiu Simion Cotoi^1,3^, Mihaela Elena Tomut^3^, Gabriel Jakobsson^6^, Maya Simionescu^2^, Felicia Antohe^2^ ‡, Alexandru Schiopu^1,5,6,7^ ‡*

^1^ Department of Pathophysiology, George Emil Palade University of Medicine, Pharmacy, Science, and Technology of Targu Mures, Targu Mures, Romania

^2^ Department of Proteomics, Institute of Cellular Biology and Pathology “Nicolae Simionescu”, Bucharest, Romania

^3^ Clinical County Hospital, Targu Mures, Romania

^4^ Department of Clinical Sciences Malmö, Lund University, Sweden

^5^ Molecular and Cellular Pharmacology – Functional Genomics, Institute of Cellular Biology and Pathology “Nicolae Simionescu”, Bucharest, Romania

^6^ Department of Translational Medicine, Lund University, Sweden

^7^ Department of Internal Medicine, Skane University Hospital, Lund, Sweden

† These authors have equally contributed to the work and share first authorship

‡ These authors have equally contributed to the work and share last authorship

**Correspondence:
*** Alexandru Schiopu, [alexandru.schiopu@med.lu.se](mailto:alexandru.schiopu@med.lu.se)

* Razvan Gheorghita Mares, [razvan.mares@umfst.ro](mailto:razvan.mares@umfst.ro)

**Supplementary Table 1.** Protein inference parameters for the 25 differentially abundant proteins associated to angiogenesis-related biological processes that were regulated by S100A8/A9 blockade.

Confidence attributes such as the number of unique peptides and the Sequest score, and the biological processes involving these proteins are presented.

| **Accession** | **Description** | **Gene symbol** | **No. of unique peptides** | **Sequest Score** | **Biological process** |
| --- | --- | --- | --- | --- | --- |
| P10107 | Annexin A1 | AnxA1 | 23 | 475.53 | angiogenesis,  sprouting angiogenesis |
| P07356 | Annexin A2 | AnxA2 | 16 | 580.93 | angiogenesis |
| O35639 | Annexin A3 | AnxA3 | 11 | 223.83 | angiogenesis,  regulation of angiogenesis,  positive regulation of angiogenesis |
| P18572-1 | Basigin | Basi | 9 | 902.2 | angiogenesis |
| P51437 | Cathelicidin antimicrobial peptide | Camp | 2 | 121.04 | angiogenesis,  regulation of angiogenesis,  positive regulation of angiogenesis |
| P60766-2 | Cell division control protein 42 homolog | Cdc42 | 9 | 553.6 | angiogenesis,  sprouting angiogenesis |
| Q9QYB1 | Chloride intracellular channel protein 4 | Clic4 | 4 | 71.6 | angiogenesis |
| P02463 | Collagen alpha-1(IV) chain | Co4A1 | 6 | 223.34 | angiogenesis |
| P08122 | Collagen alpha-2(IV) chain | Co4A2 | 3 | 91.95 | angiogenesis,  regulation of angiogenesis,  negative regulation of angiogenesis |
| P29268 | Connective tissue growth factor | Ctgf | 7 | 87.94 | angiogenesis |
| Q62165 | Dystroglycan | Dag1 | 13 | 813.31 | angiogenesis,  angiogenesis involved in wound healing |
| Q3TAS6 | ER membrane protein complex subunit 10 | Emc10 | 3 | 121.66 | angiogenesis,  regulation of angiogenesis,  positive regulation of angiogenesis |
| P61148 | Fibroblast growth factor 1 | Fgf1 | 3 | 132.05 | angiogenesis,  regulation of angiogenesis,  positive regulation of angiogenesis,  sprouting angiogenesis,  positive regulation of sprouting angiogenesis,  regulation of sprouting angiogenesis |
| Q8BTM8 | Filamin-A | Flna | 17 | 165.11 | angiogenesis |
| P11352 | Glutathione peroxidase 1 | Gpx1 | 10 | 966.72 | angiogenesis,  angiogenesis involved in wound healing |
| Q64314 | Hematopoietic progenitor cell antigen CD34 | Cd34 | 2 | 61.02 | angiogenesis,  regulation of angiogenesis,  positive regulation of angiogenesis,  angiogenesis involved in wound healing |
| Q9Z2D6-2 | Isoform B of Methyl-CpG-binding  protein 2 | Mbd2 | 6 | 87.47 | angiogenesis,  regulation of angiogenesis,  negative regulation of angiogenesis |
| Q8K4Z3 | NAD(P)H-hydrate epimerase | Apoa1bp | 3 | 187.4 | angiogenesis,  regulation of angiogenesis,  negative regulation of angiogenesis,  sprouting angiogenesis |
| O08692 | Neutrophilic granule protein | Ngp | 10 | 644.27 | angiogenesis,  regulation of angiogenesis,  negative regulation of angiogenesis |
| Q9Z126 | Platelet factor 4 | Pf4 | 4 | 126.71 | angiogenesis |
| Q99P72-2 | Reticulon-4 | Rtn4 | 17 | 857.58 | angiogenesis,  regulation of angiogenesis,  positive regulation of angiogenesis,  sprouting angiogenesis,  cell adhesion involved in sprouting angiogenesis, angiogenic sprout fusion |
| P07214 | Sparc | Sprc | 8 | 288.56 | angiogenesis,  regulation of angiogenesis,  negative regulation of angiogenesis |
| P01831 | Thy-1 membrane glycoprotein | Thy1 | 6 | 149.53 | angiogenesis |
| P39447 | Tight junction protein ZO-1 | Tjp1 | 4 | 24.16 | angiogenesis,  regulation of angiogenesis,  positive regulation of angiogenesis,  sprouting angiogenesis,  positive regulation of sprouting angiogenesis, regulation of sprouting angiogenesis |
| Q9QUI0 | Transforming protein RhoA | Rhoa | 5 | 320.76 | angiogenesis,  sprouting angiogenesis |
