# Supplementary material for: Short-term S100A8/A9 Blockade Promotes Cardiac Neovascularization after Myocardial Infarction
Source: J Cardiovasc Transl Res. 2024 Jul 15;17(6):1389–99. doi: 10.1007/s12265-024-10542-6 (PMC11634919; doi:10.1007/s12265-024-10542-6)
Supplement: Supplementary file 2 — Supplementary file2 (DOCX 46 KB) [file 12265_2024_10542_MOESM2_ESM.docx]

Short-term S100A8/A9 blockade promotes cardiac neovascularization after myocardial infarction

Razvan Gheorghita Mares^1^†*, Viorel Iulian Suica^2^†, Elena Uyy^2^, Raluca Maria Boteanu^2^, Luminita Ivan^2^, Iuliu Gabriel Cocuz^1,3^, Adrian Horatiu Sabau^1,3^, Vikas Yadav^4^, Istvan Adorjan Szabo^1^, Ovidiu Simion Cotoi^1,3^, Mihaela Elena Tomut^3^, Gabriel Jakobsson^6^, Maya Simionescu^2^, Felicia Antohe^2^ ‡, Alexandru Schiopu^1,5,6,7^ ‡*

^1^ Department of Pathophysiology, George Emil Palade University of Medicine, Pharmacy, Science, and Technology of Targu Mures, Targu Mures, Romania

^2^ Department of Proteomics, Institute of Cellular Biology and Pathology “Nicolae Simionescu”, Bucharest, Romania

^3^ Clinical County Hospital, Targu Mures, Romania

^4^ Department of Clinical Sciences Malmö, Lund University, Sweden

^5^ Molecular and Cellular Pharmacology – Functional Genomics, Institute of Cellular Biology and Pathology “Nicolae Simionescu”, Bucharest, Romania

^6^ Department of Translational Medicine, Lund University, Sweden

^7^ Department of Internal Medicine, Skane University Hospital, Lund, Sweden

† These authors have equally contributed to the work and share first authorship

‡ These authors have equally contributed to the work and share last authorship

**Correspondence:
*** Alexandru Schiopu, [alexandru.schiopu@med.lu.se](mailto:alexandru.schiopu@med.lu.se)

* Razvan Gheorghita Mares, [razvan.mares@umfst.ro](mailto:razvan.mares@umfst.ro)

**Supplementary methods**

**Animals**

For the histology experiments, female wild-type (C57BL/6) mice, 8-12 weeks of age, 20-25 g body weight, were purchased from the Cantacuzino National Research and Development Institute, Bucharest, Romania. All experimental procedures involving animals included in the histology analysis were conducted at the experimental station of “George Emil Palade” University of Medicine, Pharmacy, Science, and Technology of Targu-Mures, Romania, according to protocols approved by the Ethics Committee for Scientific Research of the University. The mice were allowed to acclimatize for at least one week before being included into the study. All animals were housed in cages that provided appropriate space with a 12-hour light-dark cycle and free access to water and regular mouse diet. The proteomic experiments included male and female wild-type (C57BL/6) mice, 8-12 weeks of age, 20-25 g body weight, bread at the animal care facility of the Institute for Cellular Biology and Pathology “N. Simionescu”, Bucharest, Romania. All proteomic experiments have been approved by the Ethics Committee of the Institute and by the National Sanitary Veterinary and Food Safety Authority (no. 425/22.10.2018) in accordance with Directive 2010/63 of European Union. Adequate environmental conditions (temperature, humidity and ventilation) were ensured according to the current guidelines for the accommodation and care of laboratory animals. The study was conducted in accordance with the National Institutes of Health guide for the care and use of Laboratory animals (NIH Publications no. 8023, revised 1978), and Romanian Law no. 471/2002.

**MI induction**

MI was induced as previously described [12,13] by permanent left coronary artery (LAD) ligation. Briefly, mice were placed in prone position on a heated surgical board to maintain body temperature at around 37ºC and were continuously anaesthetized with 2-3% isoflurane combined with 0.5 L/minute oxygen administered through a non-invasive inhalation system (EZ-SA800 Single Animal System, Philadelphia, USA). The fur was removed with a standard trimmer for small animals and the skin was cleaned with water and thereafter with betadine pads. A small skin incision (1-1.2 cm) was made on the left side of the chest and the heart was exposed through a small opening in the thorax at the level of the fourth intercostal space. Next, the LAD was visually located and ligated at approximately 2-3 mm from the origin by using a 6.0 silk suture. The ligation was considered successful if the anterior wall of the left ventricle turned pale. After ligation, the heart was immediately placed back into the thorax, the pneumothorax was manually evacuated by gentle bilateral side pressure and the skin was closed with 6.0 Prolene suture. The sham group underwent the same surgical procedure except for the LAD ligation. At experiment termination, the animals were euthanized under general anesthesia induced by i.p. injection of a ketamine-xylazine (100/20 mg/kg body weight) solution and the hearts were collected for histological and proteomics analysis.

**Experimental groups and treatments**

Immediately after MI surgery, the mice were randomly assigned into 2 groups and treated with PBS (MI group) or with 30mg/kg of the small-molecule S100A8/A9 blocker ABR-238901 (ABR) diluted in PBS (MI+ABR group). ABR-238901, a gift from Active Biotech AB (Lund, Sweden), inhibits the binding of S100A8/A9 to its receptors [8]. For the histology experiments, MI was confirmed at harvest by Hematoxylin and eosin (H&E) and Trichrome-Masson stains, and only surviving mice with confirmed infarction were included in the S100A9 and CD31 analysis. The final MI mouse groups included 4-7 mice and the sham groups included 3 mice, as specified in the figure legends. Separate mouse groups were sacrificed at 1-, 3- and 7-days post-MI and the hearts were collected for immunohistochemical analysis. Mice harvested on day 1 post-MI received one i.p. injection of either PBS or ABR, administered immediately after MI. All other mouse groups received a total of three i.p. injections of PBS or ABR administered at the time of the MI, and repeated after 24 and 48 hours. For the proteomics analysis, MI induction was confirmed before harvest by echocardiography. We applied strict inclusion/exclusion criteria and excluded from the proteomics analysis animals with left ventricular ejection fraction >40%. The final MI mouse groups for the proteomics study included 4-5 mice that survived until day 7 and had a left ventricular ejection fraction <40%. Three mice have been included in the sham group, as specified in the figure legends. The infarcted regions of cardiac left ventricle below the ligature were harvested at 7-days post-MI and homogenized with TRIzol Reagent (Sigma-Aldrich, MO, USA) for subsequent proteomic assays.

**Immunohistochemistry**

The expression of S100A9 and of the endothelial cell marker CD31 in cardiac tissue was assessed by immunohistochemical staining. At harvest, the hearts were thoroughly perfused with PBS to remove intracoronary blood. The hearts were fixed overnight with 10% formalin, embedded in paraffin, and serially sectioned in 4 μm-thick sections collected at 300 µm intervals along the transversal axis, from the apex to the level of the ligature. Antigen retrieval was performed in a target retrieval solution (Leica Microsystems, Germany) using microwave heating for 20 min, and endogenous peroxidases were quenched with 3% H_2_O_2_ for 10 min. The sections were blocked with Protein Block Serum-Free solution (Dako, CA, USA) and incubated with monoclonal IgG rabbit-anti mouse anti-CD31 primary antibody (1:100 dilution) (PECAM-1, clone D8V9E,) or a monoclonal IgG rabbit anti-mouse anti-S100A9 primary antibody (1:800 dilution) (clone D3U8M) overnight at 4ºC. For the detection step, the sections were incubated for 1h at room temperature with BrightVision Goat-Anti Rabbit IgG (H+L)-Poly-HRP Biotin-free (ready-to-use) secondary antibody (Immunologic, Amsterdam, The Netherlands). The signal was amplified and detected using 3,3′-Diaminobenzidine (DAB) and the slides were counterstained with hematoxylin according to the manufacturer’s instructions. The primary antibodies against S100A9 and CD31 were purchased from Cell Signaling Technology (Danvers, MA, USA).

**Measurement of myocardial S100A9 infiltration and CD31 abundance**

We performed histological analyses of heart sections collected at 1-, 3- and 7-days post-MI. All images were taken with an Axio Imager Z2 microscope with a color Axiocam 506 camera and processed using the ZenPro 3.2 software (all Zeiss, Germany) and QuPath version 0.3.0 (https://qupath.github.io). The S100A9 and CD31 presence was quantified as a percentage of the entire left ventricle (LV) area, and of the infarcted/border zone and the remote myocardial area separately. For each marker, we analyzed myocardial tissue sections collected from 5-6 different levels along the transversal axis of the heart and averaged the values.

**Liquid chromatography – tandem mass spectrometry (LC‐MS/MS) and statistical analysis**

The proteomic analysis was performed by liquid chromatography and mass spectrometry as previously described [14]. From each experimental condition (Sham, MI and MI+ABR), 50μg of proteins were extracted from the infarcted regions of cardiac left ventricle below the ligature (MI groups) or from the whole heart (Sham group). The proteins were purified by acetone precipitation, before carbamidomethylation of the cysteine residues that was done with 20mM dithiothreitol (in a buffer containing 8M urea, 0.1M Tris‐HCl and 0.1mM EDTA, pH 8.8, 1h under agitation) and 80mM iodoacetamide (in 0.1M Tris‐HCl and 0.1mM EDTA buffer, in the dark under agitation). The protein samples were incubated overnight at 37⁰C with trypsin (1:20 w/w) for proteolysis. The resulted peptides were desalted with C18 columns, concentrated and injected (1μg/sample, in technical triplicates) in the Easy nLC II liquid chromatograph (Thermo Fisher Scientific, Waltham, MA, USA). The peptides were first loaded on a pre-column (Thermo Scientific Easy Column-2 cm length, 100μm inner diameter, 5μm particle size, 100Å pore size) and separated on the 10cm EASY analytical column (Thermo Scientific-75μm inner diameter, 3μm particle size, 100Å pore size) using a 300nL/min, 3–25% solvent B gradient (0.1% formic acid in acetonitrile, while solvent A was 0.1% formic acid in water) over a 120 min period. The spectra of the separated peptides were acquired by the LTQ‐Velos Pro Orbitrap hybrid mass spectrometer (Thermo Scientific), which was operated in a top 12 data‐dependent configuration (DDA), at 60k resolution for a full scan across the 350–1700m/z domain. Collision‐induced dissociation was enabled for parent ion fragmentation, in order to obtain MS2 spectra.

The mass spectrometry raw data were analyzed with the Proteome Discoverer 2.4 software (Thermo Scientific), while for protein inference, UniProtKB/Swiss‐Prot mouse reference protein database (UP000000589 Proteome ID, v.04.2019) was used. Two maximum miss-cleavages were allowed, cysteine carbamidomethylation was set as fixed modification, and oxidation of methionine and deamidation of asparagine and glutamine were selected as variable modifications. The target protein false discovery rate (FDR) was set below 0.05. Label‐free relative protein quantitation on the precursor level was performed using Proteome Discoverer 2.4. ANOVA hypothesis test was used to determine the statistical significance associated with the protein ratio calculation. The Benjamini–Hochberg FDR-based correction was used for p-value adjustment. Out of all identified proteins, we selected only the proteins that were significantly up- or down- regulated by >1.25-fold in the MI+ABR/MI comparison. These proteins were used for the “Biological processes” Gene Ontology analysis using g:Profiler (version e106_eg53_p16_65fcd97, database update 18.05.2022) to reveal the angiogenesis-related biological processes that were over-represented. An over-represented biological process was considered to be statistically significant if the Benjamini–Hochberg FDR-based corrected p-value was < 0.05.

**Endothelial cell apoptosis assay in-vitro**

Human Umbilical Vein Endothelial Cells (HUVECs, Thermo Fisher Scientific, Waltham, MA) were cultured in endothelial cell basal medium (Promocell GmbH, Heidelberg, Germany) containing 2% serum, growth supplements (human recombinant Epidermal growth factor and Fibroblast growth factor) and 1% antibiotics (Invitrogen, CA, USA). The cells included in the experiments have undergone between 4 and 8 passages. HUVECs were dissociated using Accutase (Gibco, USA) to obtain a cell suspension, were seeded into 96-well plates at a density of 3x10^4^ cells per well and allowed to adhere overnight. Thereafter, the cells were treated with 5 μg/ml or 10 μg/ml recombinant human S100A8/A9 in the presence or absence of 100μM ABR-238901 for 24 h in 0.5% low serum medium. Untreated cells and cells treated with 100μM ABR-238901 alone served as controls. To assess apoptosis induction, we measured the levels of active caspase-3/7 in cell lysates by using the Caspase-Glo 3/7 kit (Promega, Wisconsin, USA), according to manufacturer’s instructions.

**Statistical analysis**

All data from the immunohistochemistry experiments are expressed as mean ± standard deviation (SD). The Shapiro-Wilk test confirmed the normality of data distribution for groups with low numbers of replicates, allowing parametric tests to be used for the statistical analysis. Comparisons among three groups were performed using one-way ANOVA with Fisher’s LSD post-hoc test. Comparisons between two groups were performed with Student’s T-test. The GraphPad Prism 6.0 software (GraphPad, CA, USA) was used for the statistical analysis. A p-value <0.05 was considered to be statistically significant.
